# EVALI Vaping Liquids Part 1: GC-MS Cannabinoids Profiles and Identification of Unnatural THC Isomers

**DOI:** 10.3389/fchem.2021.746479

**Published:** 2021-09-14

**Authors:** Laura A. Ciolino, Tracy L. Ranieri, Jana L. Brueggemeyer, Allison M. Taylor, Angela S. Mohrhaus

**Affiliations:** Forensic Chemistry Center, US Food and Drug Administration, Cincinnati, OH, United States

**Keywords:** EVALI, vaping liquids, e-cigarettes, tetrahydrocannabinol, THC isomers, THC distillates, THC concentrates, GC-MS

## Abstract

Tetrahydrocannabinol (THC)-containing products played a major role in the 2019 US nationwide outbreak of pulmonary lung illness associated with e-cigarettes or vaping liquids (EVALI). Due to the severity of the illness which resulted in 68 deaths, a comprehensive identification of the components in the vaping liquids was required. Our laboratory received over 1000 vaping liquid products for analysis including hundreds of vaping products from EVALI patients. In this work, we present the results for the GC-MS identification of the cannabinoids from a large subset of ca. 300 Cannabis-based vaping liquids, with emphasis on the identification of a series of unnatural THC isomers. GC-MS analysis was conducted using a validated, published method in which the cannabinoids were identified as the trimethylsilyl derivatives after separation on a commercial 35% silphenylene phase. Δ^9^- Tetrahydrocannabinol is the naturally occurring THC isomer found in the Cannabis plant, and was found in the majority of the vaping liquids. However, we also identified the presence of one or more additional THC isomers in many of the vaping liquids including Δ^8^-tetrahydrocannabinol, Δ^6a,10a^–tetrahydrocannabinol, Δ^10^-tetrahydrocannabinol, and exo-tetrahydrocannabinol. Significant or major amounts of unnatural THC isomers were found in over 10% of the THC vaping liquids, with lesser amounts found in another 60% of the vaping liquids. Exposure of the Cannabis source materials (such as marijuana concentrates or converted hemp materials) to chemical and thermal treatments during manufacturing, is proposed as the primary cause for the THC isomerizations.

## Introduction

In 2019, there was a nationwide outbreak of pulmonary lung illness associated with the use of e-cigarettes or vaping products (EVALI). The outbreak resulted in over 2,800 hospitalizations and 68 deaths (US Centers for Disease Control Office on Smoking and Health National Center for Chronic Disease Prevention and Health Promotion, 2020). After months of investigation, the US Centers for Disease Control (CDC) concluded that tetrahydrocannabinol (THC)-containing products played a major role in the outbreak (Lozier et al., 2019), and that the presence of the additive vitamin E acetate in the products was also strongly linked to the outbreak (Blount et al., 2020). While vitamin E acetate was strongly implicated in the outbreak, the CDC also concluded that the contribution of other chemicals in either THC or non-THC vaping liquid products could not be ruled out in some EVALI cases (US Centers for Disease Control Office on Smoking and Health National Center for Chronic Disease Prevention and Health Promotion, 2020).

In 2019 and 2020, our laboratory received more than 1000 Cannabis-based vaping products for analysis, including over 500 products from EVALI patients, as well as unused products from various sources. Our laboratory was charged with a comprehensive identification of the components in the vaping liquids which included the cannabinoids, and additives such as cutting agents and diluents. A full array of qualitative analysis was conducted on the vaping liquids including Fourier transform infrared (FT-IR) and Raman spectroscopy, gas chromatography-mass spectrometry (GC-MS), liquid chromatography-high resolution mass spectrometry (LC-HRMS), and direct analysis in real time-high resolution mass spectrometry (DART-HRMS).

GC-MS is ideal for the analysis of vaping liquids because they are largely formulated using volatile or semi-volatile substances. In Part 1 of this work, we report on the GC-MS identification of the cannabinoids in the vaping fluids, with prominence given to the identification of a series of unnatural THC isomers. In Part 2^1^, we report on the GC-MS identification of several vaping liquid additives, with confirmation of selected additives using LC-HRMS. The vaping liquids presented in this work represent a large subset (ca. 300) of the Cannabis vaping liquids which were analyzed by our laboratory, and include over 150 vaping liquids from EVALI patients. Given the scope and severity of the EVALI illnesses, this work provides valuable information towards a more complete understanding of vaping liquid compositions, and on analytical approaches for characterizing vaping liquids.

The cannabinoids discussed in this work will include Δ^9^-tetrahydrocannabinol (hereafter “d9THC”), Δ^8^-tetrahydrocannabinol (d8THC), 9R-Δ^6a,10a^ - tetrahydrocannabinol (9R-d6a,10aTHC), 9S-Δ^6a,10a^ - tetrahydrocannabinol (9S-d6a,10aTHC), 6aR,9R- Δ^10^-tetrahydrocannabinol (6aR,9R-d10THC), 6aR,9S- Δ^10^-tetrahydrocannabinol (6aR,9S-d10THC), exo-tetrahydrocannabinol (exoTHC), Δ^9^-tetrahydrocannabinolic acid A (THCA), Δ^9^-tetrahydrocannabivarin (d9THCV), Δ^8^-tetrahydrocannabivarin (d8THCV), Δ^6a,10a^-tetrahydrocannabivarin (d6a,10aTHCV), cannabidiol (CBD), cannabidivarin (CBDV), cannabinol (CBN), cannabigerol (CBG), and cannabichromene (CBC).

## Materials and Methods

### Standards, Solvents, and Reagents

d9THC, d8THC, exoTHC, THCA, CBD, CBN, CBG, CBC, d9THCV, and CBDV were obtained as certified 1.0 mg/ml stock solutions (acetonitrile or methanol) from Cerilliant Corporation (Round Rock, TX). 9R-d6a,10aTHC, 9S-d6a,10aTHC, 6aR,9R-d10THC, 6aR,9RS-d10THC, and d8THCV were obtained as 1.0 or 5.0 mg/ml stock solutions (acetonitrile) from Cayman Chemical Company (Ann Arbor, MI, all purities ≥95%). Olivetol (95%) was obtained from Sigma Aldrich (St. Louis, MO), and a stock solution was prepared in ethanol. For analysis, aliquots (10—200 μL) of the standard stock solutions were taken for trimethylsilyl (TMS) derivatization in the same manner as the samples (see *GC-MS Analysis* Section).

Ethanol (200 proof, USP/ACS grade) was obtained from Sigma Aldrich (St. Louis, MO). Acetonitrile (HPLC grade) and pyridine (certified ACS) were obtained from Fisher Scientific (Waltham, MA). BSTFA reagent [99:1 N,O-bis(trimethylsilyl)trifluoroacetamide: trimethylchlorosilane] was obtained from Regis Technologies (Morton Grove, IL). Deionized water (18 Mohm) was obtained from a Millipore filtration system fed by a service deionized water source.

### Preparation of Sample Concentrates

Used and unused vaping cartridges were received. For used cartridges, the remaining vaping liquid amounts ranged from residues to almost full cartridges. Full cartridges contained up to 1 g or 1 ml of vaping liquid. Prior to sampling for analysis, the vaping liquid contents were transferred from the cartridges or vaping devices to 2 ml autosampler glass vials (Water Corp.) for storage as follows. The receiving vial was placed in the bottom of a 15 ml conical bottom centrifuge tube (Falcon brand). A 5 ml plastic disposable pipet tip (Rainin RC-L5000) was placed into the receiving vial with the pipet tip end pointed downward. The vaping cartridge or device was disassembled, and the open end was placed into the top end of the pipet tip so as to allow flow of the vaping liquid out of the device through the pipet tip and into the receiving vial. The entire assembly was then placed in a centrifuge and spun until transfer of the vaping liquid into the receiving vial was complete (3–5 min). An IEC clinical centrifuge (dial setting 3) or Thermo Sorvall Legend RT centrifuge (2000 rpm) was used. The amount of vaping liquid recovered from unused cartridges was in the range 0.7—1.0 g, and the amount of vaping liquid recovered from used cartridges was in the range 0.002—0.9 g. Based on visual observation of their flow behaviors, the vaping liquids we encountered typically consisted of medium to high viscosity liquids.

Due to the limited sample amounts for many of the vaping liquids, and the difficulty of sampling viscous liquids without considerable waste, an initial concentrated extract of the vaping liquid (referred to as the “sample concentrate”) was prepared in 95% ethanol. Sample concentrates were prepared in 1.0 ml or 4.0 ml glass sample vials, with vaping liquid sample weights typically in the range 10—100 mg. Solvent volumes were typically in the range 0.5–1.0 ml, resulting in finished sample concentrates generally in the range 20—100 mg vaping liquid per ml. After addition of solvent, the sample vial was capped and then briefly warmed on a hot plate as needed to speed dissolution of the vaping liquid (one or 2 min, ≤ 100°C). After dissolution of the vaping liquid, the sample vial was mixed on a vortexer to produce a homogeneous solution. Once prepared, aliquots of the sample concentrate were taken as described below for GC-MS qualitative analysis or HPLC-DAD quantitative analysis of the Cannabis cannabinoids. When sufficient vaping liquid was available, duplicate preparations of sample concentrates were made, and analyzed as described.

### GC-MS Analysis

GC-MS analysis was conducted using a validated method (Ciolino et al., 2018a). The sample concentrates were mixed on a vortex mixer prior to sampling for GC-MS analysis. A dilution of the sample concentrate was made directly into a GC vial using acetonitrile as the diluent, with sample concentrate aliquot volumes generally in the range 25—100 μl and a finished volume of ca. 1.0 ml after addition of acetonitrile. A portion (generally in range 50–200 μL) of the diluted sample was transferred to a GC vial for derivatization. The solvent was evaporated under a stream of dry air on a Pierce Reacti-therm block (nominal block temperature 70–80°C). 200 μl pyridine and 200 μl BSTFA reagent were added to the vial, the vial was capped, mixed, and incubated for 30 min (70–80°C). Analysis was carried out using an Agilent 7890B 70 eV EI GC–MS system with 5977B MS detector. The column was a 30 m Restek Rxi-35Sil MS (35% silphenylene) with 0.25 mm internal diameter and 0.25 μm film thickness. Injection volume was 1 μL (splitless) with an injection port temperature of 250°C. The carrier gas was helium with a flow rate of 0.8 ml/min (constant flow mode). Oven program was as follows: initial temperature 60°C with 0.5 min hold, first ramp 25°C/min to 220°C, hold for 10 min, second ramp 10 ^o^C/min to 300°C, with a final hold time of 15 min (run time 39.9 min). Transfer line temperature was 280°C. Solvent delay was 7.0 min, and MS acquisition used full scan mode with mass range 40–600 amu.

### HPLC-DAD Analysis

HPLC-DAD analysis was conducted using a validated method (Ciolino et al., 2018b). The sample concentrates were mixed on a vortex mixer prior to sampling for HPLC-DAD analysis. Further dilutions of the vaping liquid sample concentrates were made using 95% ethanol either into volumetric flasks, or directly into LC vials to bring the final cannabinoid concentrations into the linear ranges (generally less than 500 μg/ml). Because most vaping liquids were completely soluble in the 95% ethanol, the preparations were only filtered (using 0.45 micron nylon membrane filters) if there was sufficient volume, or in rare cases of seeing precipitates. Analysis was conducted using Agilent 1100, 1200, or 1260 HPLC-DAD systems. Separations were carried out using MacMod ACE 5 C18-AR analytical columns (5 μm, 4.6 mm ID x 250 mm length). The mobile phase comprised 66:34 acetonitrile: 0.5% acetic acid (no pH adjustment, nominal pH 2.9). The injection volume was 25 μl, flow rate 1.0 ml/min, and run time 60 min. Detection wavelength was 240 nm. Chromatographic peak spectra were obtained over the range 190—400 nm.

## Results and Discussion

The commercial production of highly concentrated marijuana extracts or resins intended for use in products such as vaping liquids has been reported (Finley et al., 2016; WHO Expert Committee on Drug Dependence, 2018a; Finley and Mckee, 2019; Ko and Hughes, 2019). Finished concentrates with d9THC purities in the range 80—99% w/w have been described (Finley et al., 2016; Finley and Mckee, 2019; Ko and Hughes, 2019). Prior to use in a vaping liquid, the marijuana extracts are typically dewaxed (WHO Expert Committee on Drug Dependence, 2018a; Ko and Hughes, 2019), and may undergo additional processing steps including distillation, and decarboxylation of the acidic cannabinoids (Finley et al., 2016; Finley and McKee, 2019; Ko and Hughes, 2019). The processed marijuana extracts are frequently formulated with additives 1 to produce vaping liquids. Both high d9THC marijuana concentrates and finished vaping liquids are also produced on the black market or in clandestine labs (US Drug Enforcement Administration Diversion Control Division, 2020a; 2020b)**.** Some end users may formulate their own vaping fluids.

**FIGURE 1:**
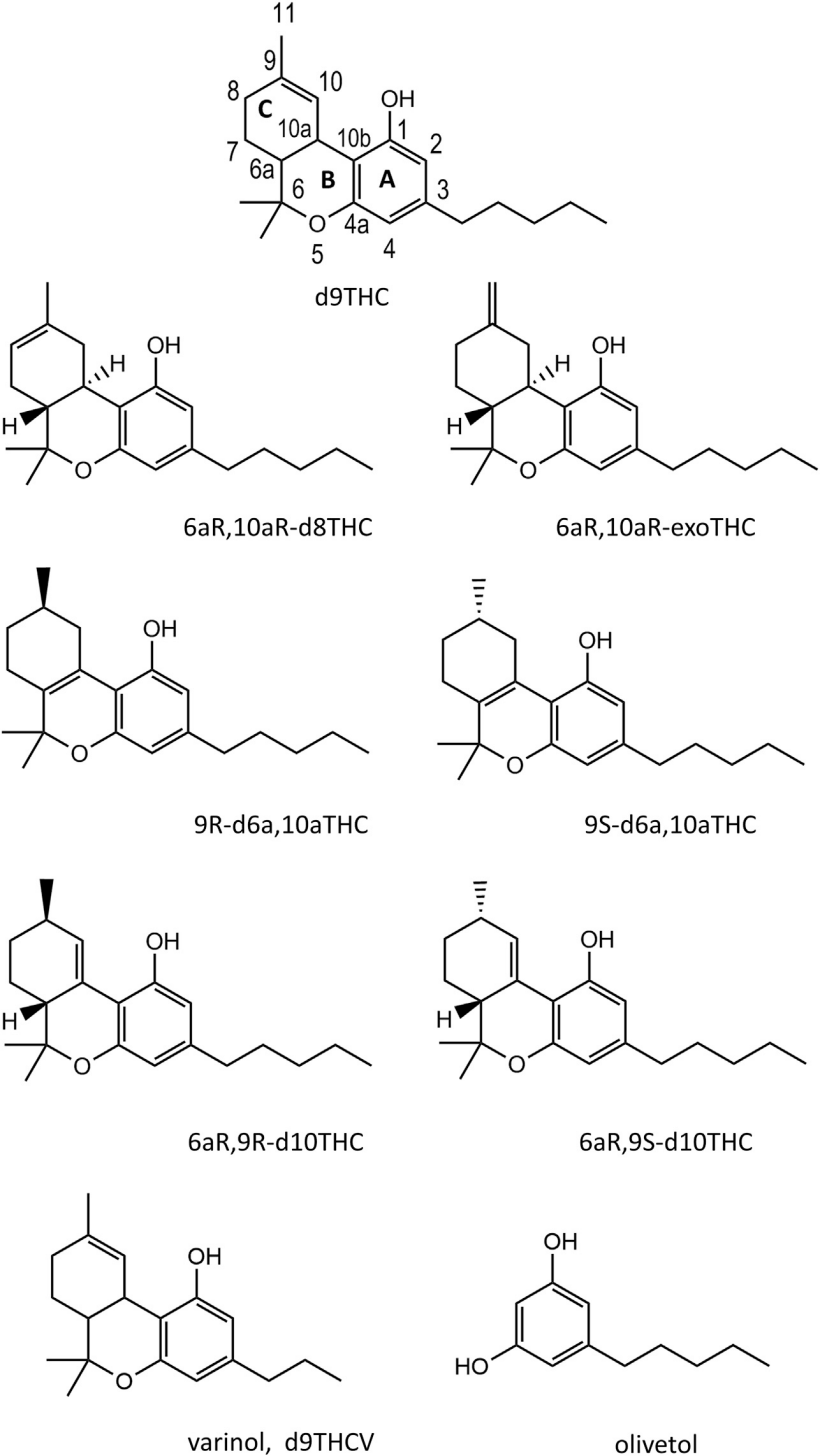
Chemical structures for seven THC isomers, a representative varinol cannabinoid (d9THCV, lowest left structure), and olivetol (lowest right structure).

The majority of the vaping liquids we analyzed contained substantial levels of d9THC in the liquids, or d9THC represented the predominant cannabinoid in the cannabinoids profile. Using the validated HPLC-UV method (Ciolino et al., 2018b), we determined levels of up to 80% w/w d9THC, and frequently above 50% w/w, in vaping liquids in which no additives were identified (unpublished data). Despite this commonality, we saw a variety of cannabinoids profiles for the vaping liquids, making it challenging to group them into simple categories. The most striking result was the occurrence of many high THC vaping liquids in which unnatural THC isomers were encountered, including d8THC, 9R- or 9S-d6a,10aTHC, 6aR,9R-d10THC, 6aR,9RS-d10THC, and exoTHC (see Figure 1, THC isomer structures). Among a tally of 214 high THC vaping liquids, we found 58 vaping liquids with unnatural levels of d8THC, 26 vaping liquids with significant or major levels of the d6a,10aTHC/d10THC isomers, 138 vaping liquids with some or minor levels of the d6a,10aTHC/d10THC isomers, and only 38 vaping liquids which contained d9THC as the only THC isomer.

The vaping liquids also contained other naturally occurring cannabinoids such as CBD, CBN, CBG, and CBC, as well as the varinol cannabinoids. The varinol cannabinoids, such as d9THCV and CBDV, are analogous in structure to the main cannabinoids except that the alkyl side chain on the resorcinol ring is a propyl chain instead of a pentyl chain (Hanuš et al., 2016, see also Figure 1, d9THCV structure). As discussed later, the presence of varinol cannabinoids in the vaping liquids argues against a synthetic source for the THC raw materials. Among another tally of 299 vaping liquids, only 18 contained both d9THC and THCA. A few of the vaping liquids contained CBD or CBN as the predominant cannabinoid. The remainder of the work presented here will focus on the details of the THC isomer profiles which were encountered in the vaping liquids.

### Vaping Liquid THC Isomer Profiles

In the figures and discussion which follow, we have organized the vaping liquid examples according to the one or two most predominant cannabinoids or other distinctive features. All GC-MS chromatograms are for the “derivatized sample preparations”, in which the cannabinoids have been converted to their fully trimethylsilyated derivatives (d9THC monoTMS, CBD diTMS, THCA diTMS, etc.). However, for purposes of simplicity in the discussion, the cannabinoids will be referred to as the parent compounds. At the time of the EVALI crisis, our validated HPLC-DAD method for cannabinoids quantitation addressed a total of 13 cannabinoids which included only two of the THC isomers, d9THC and d8THC. HPLC-DAD retention times were subsequently established for the exoTHC, d6a, 10aTHC and d10THC isomers, and showed that these THC isomers did not coelute with either d9THC or d8THC. However, the d6a, 10aTHC and d10THC isomers were only sufficiently resolved from one another to allow for a qualitative assessment, and not for strict quantitation. Moreover, only qualitative not quantitative reference standards for the d6a, 10aTHC and d10THC isomers were commercially available. HPLC-DAD quantitation was also not conducted for exoTHC, as it was either not detected, or found in only minor amounts. Hence, in this work, quantitative results are provided only for d9THC, d8THC, and CBD. However, as will be demonstrated, the GC-MS peak area percentages (PAPs) for the THC isomers provide reasonable estimates of the relative amounts of all the THC isomers in the vaping liquids. We analyzed vaping liquids from both unused and used vaping product cartridges. Many labels for vaping cartridges declare fill weights or volumes of 1 g or 1 ml. In our work with emptying and weighing the vaping liquid contents from previously unused vaping cartridges, we obtained weights in the range 0.7–1 g. For the used cartridges, the remaining amount of vaping liquid recovered by our laboratory ranged from residues (less than 10 mg) to near the nominal fill weights. For each example we note the amount of vaping liquid recovered from the cartridge (see Table 1, second last column).

**TABLE 1:** GC-MS peak area percentages (PAPs) for cannabinoids and olivetol in vaping liquids and bulk THC distillates.

Vaping			d6a,10a	6aR,9R-	6aR,9S-					Amount	
Liquids	d9THC	d8THC	THC	d10THC	d10THC	exoTHC	CBN	CBD	Olivetol^a^	Recov.(g)^b^	Additives^c^
VL#1	89	ND	ND	ND	ND	ND	7.0	3.8	ND	0.26	none ID
VL#2	94	ND	ND	ND	ND	ND	6.0	0.28	ND	0.028	none ID
VL#3	44	36	ND	ND	ND	*trace*	9.8	10	2.2	0.22	SAIB
VL#4	17	69	ND	ND	ND	1.0	4.1	9.6	6.6	0.044	MCT/SAIB
VL#5	16	66	ND	ND	ND	0.79	3.3	14	3.7	0.38	VEA
VL#6	66	11	2.5	0.83	0.39	ND	6.5	13	0.29	0.76	none ID
VL#7	66	16	7.2	0.97	0.58	ND	9.5	0.67	ND	0.42	none ID
VL#8	12	64	8.5	2.1	1.2	3.4	9.1	0.19	0.13	0.067	none ID
VL#9	9.5	69	9.4	1.6	0.98	2.6	7.1	ND	0.33	0.33	VEA
VL#10	6.1	72	13	2.0	1.1	2.2	3.8	ND	0.24	0.91	PEG
VL#11	1.4	79	7.8	1.3	0.75	2.9	7.0	ND	0.23	0.19	MCT/PEG
VL#12	33	1.4	38	10	4.7	ND	9.6	3.2	0.11	0.75	VEA/MCT
VL#13	51	3.0	21	11	4.6	ND	8.7	0.89	0.041	0.86	VEA
VL#14	43	0.4	33	7.51	2.7	ND	13	0.31	0.020	0.014	MCT/PEG
Distillates											
DST#1	76	0.68	9.6	6.2	2.1	ND	3.6	1.8	trace	NA	NA
DST#2	7.8	60	20	3.2	1.5	2.0	4.8	0.19	0.17	NA	NA

a Olivetol peak areas not included in peak area percentage calculations for cannabinoids.

b Weight of vaping liquid recovered from vaping cartridge or device.

c Additives identified in vaping liquids: SAIB-sucrose acetate isobutyrate; MCT-medium chain triglycerides oil; VEA-vitamin E acetate; PEG-polyethylene glycol; none ID-no additives identified per protocol (see Part 2).

ND-not detected; NA-not applicable.

Figure 1 shows the structures for seven THC isomer reference standards which were used in the analysis of the vaping liquids. The structure for d9THC shows the ring letter and numbering scheme. The THC isomers vary both with respect to the position of the double bond in the terpenoid ring (C ring), and the stereoisomeric forms. ExoTHC (Δ^9,11^-tetrahydrocannabinol) is the exception with the double bond being present outside the terpenoid ring. The diastereomeric form for the d9THC standard is not depicted in the figure but was the 6aR,10aR-d9THC isomer, which corresponds to the natural form found in the Cannabis plant (Hanuš et al., 2016). The d8THC and exoTHC reference standards were also the 6aR, 10aR isomers. Standards of both enantiomers of d6a, 10aTHC (9R and 9S), and two diastereomers of d10THC (6aR, 9R and 6aR,9S), were used.

**FIGURE 2:**
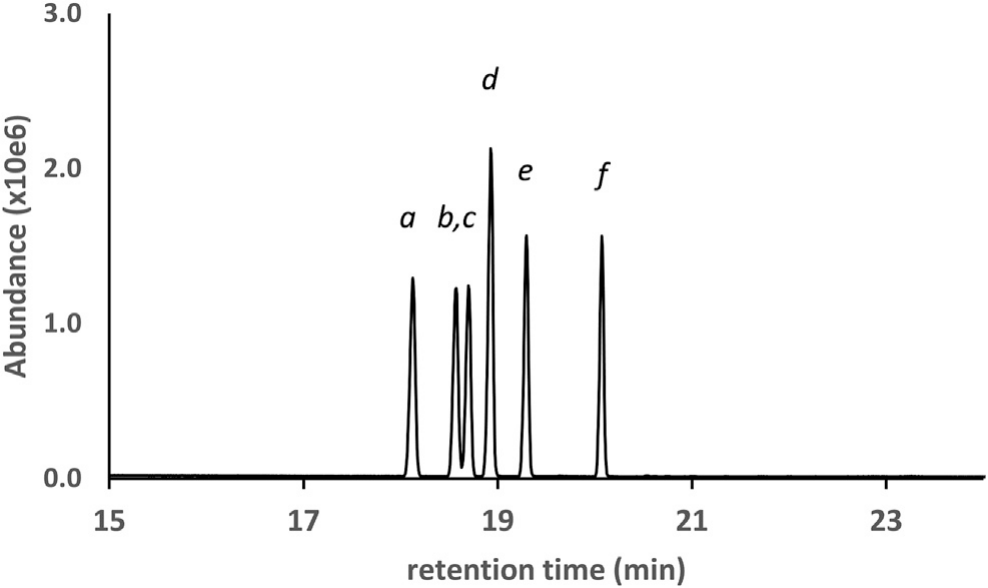
GC-MS chromatogram for a 25 μg/ml standard mix of seven positional and stereoisomeric THC monoTMS isomers. Peak labels: 6aR,10aR-d8THC(*a*); 6aR,10aR-exoTHC(*b*); 6aR,10aR-d9THC(*c*); coeluted 9R- and 9S- d6a,10aTHC(*d*); 6aR,9S-d10THC(*e*); 6aR,9R-d10THC(*f*). See text for discussion.

Figure 2 shows the GC-MS chromatogram for a 25 μg/ml standard mix of the monoTMS derivatives of the seven THC isomers. All of the positional isomers were resolved, as was the diastereomeric pair 6aR,9R- and 6aR,9S-d10THC. The only unresolved isomers were the enantiomeric pair 9R- and 9S- d6a,10aTHC, which showed complete coelution (peak label *d*). These results are typical for GC-MS analysis conducted under achiral conditions. Since the 9R- and 9S- enantiomers of d6a, 10aTHC were not resolved, further discussion will only refer to “d6a,10aTHC” for this THC isomer.

**FIGURE 3:**
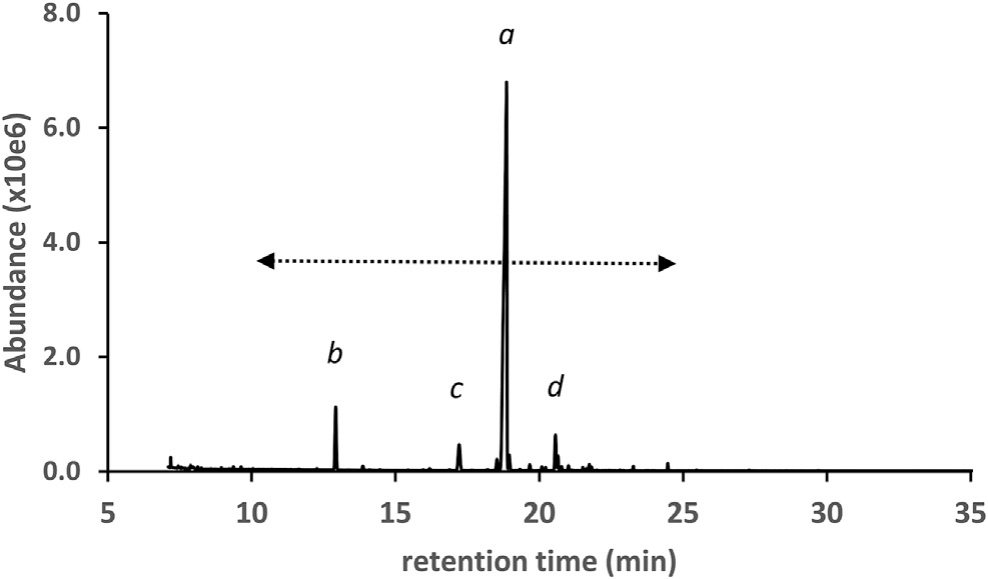
GC-MS chromatogram for a cannabis vaping liquid in which the predominant cannabinoid was d9THC(*a*), and other cannabinoids include CBD, CBG, and CBN (peak labels *b*, *c*, and *d*, respectively). The cannabinoid TMS derivatives retention range (ca. 10–25 min) is indicated by the double arrow.

Figure 3 shows the GC-MS chromatogram for a high d9THC potency vaping liquid (VL#1, Table 1) in which the predominant cannabinoid was d9THC (peak label *a*), and no additives were detected. The d9THC level in the vaping liquid was determined at 78% w/w (HPLC-DAD). Other cannabinoids identified include CBD, CBG, and CBN (peak labels *b*, *c*, and *d* respectively), and very low levels of d9THCV and CBC (peaks not visible on current scale). No d8THC or other THC isomers were identified in this vaping liquid. The figure also shows the cannabinoids retention range (10–25 min) for the current method as indicated by the double arrow (<---->). While the presence of any of the minor cannabinoids may indicate a natural Cannabis source, the presence of the varinol d9THCV in the vaping liquid is taken as stronger evidence for a plant source. The varinol series of cannabinoids are frequently found at much lower levels in Cannabis plants relative to the main cannabinoids (Hillig and Mahlberg, 2004). The presence of the varinol d9THCV, which contains a propyl side chain on the resorcinol ring (Figure 1, lowest left structure), would not be expected in any d9THC synthetic schemes, as d9THC contains a pentyl side chain in this position (Figure 1, highest center structure).

**FIGURE 4:**
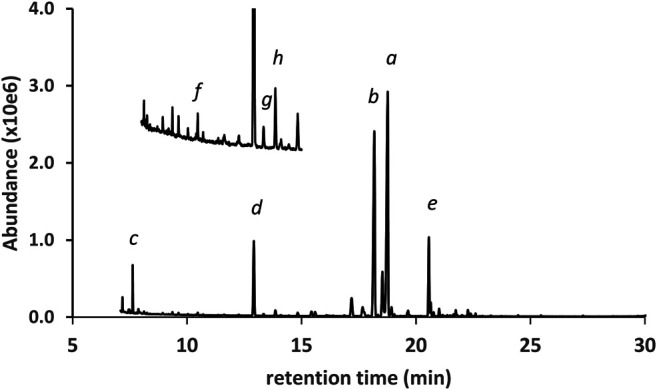
GC-MS chromatogram for a cannabis vaping liquid in which the predominant cannabinoids were d9THC(*a*) and d8THC(*b*), with lower levels of CBD(*d*) and CBN(*e*). A minor level of exoTHC (*not labeled*, peak between peaks *a* and b), and an elevated level of olivetol(*c*), were observed. Three corresponding varinols were identified (expanded scale and offset): CBDV(*f*), d8THCV(*g*), d9THCV(*h*).

Figure 4 shows the GC-MS chromatogram for a vaping liquid (VL#3, Table 1) in which the predominant cannabinoids were d9THC and d8THC (peak labels *a* and *b*, respectively). The levels of d9THC, d8THC, and CBD (peak label *d*) were determined (HPLC-DAD, %w/w) as 17, 11, and 2.2%, respectively. A minor amount of exoTHC and an elevated level of olivetol (peak label c, see also Figure 1, lowest right structure) were observed. The olivetol level was considered elevated based our prior experience with THC vaping liquids in which we have not detected olivetol, or only observed trace amounts. Low levels of the varinols CBDV, d8THCV, and d9THCV (peak labels *f*, *g*, and *h*, respectively) were also found. Again, the presence of the varinols in this vaping liquid is evidence for a natural plant source with isomerization of d9THCV to d8THCV occurring in parallel to the isomerization of d9THC to d8THC. However, the high level of d8THC is unnatural and cannot be attributed to typical processing methods for Cannabis plants. Only minor levels of d8THC have been reported in some historical studies of processed hashish or marijuana materials, with the d8THC representing one percent or less of the combined d9THC and d8THC amounts (Mechoulam, 1970). It is unclear whether minor levels of d8THC are present in unharvested Cannabis plants or if d8THC forms during plant processing steps such as the extraction or isolation of other cannabinoids. Chemical mechanisms for the conversion of either d9THC or CBD into d8THC during isolation procedures have been proposed (Hanuš et al., 2016).

Duffy et al. (2020) also reported finding unnatural amounts of d8THC in vaping liquids associated with the EVALI outbreak. The unnatural level of d8THC shows that the Cannabis material was most likely subjected to chemical and/or thermal treatments to cause substantial conversion to d8THC. Long time Cannabis researcher Mechoulam reported an early procedure for the isomerization of d9THC to d8THC which was conducted in the presence of a strong organic acid such as p-toluenesulfonic acid (Mechoulam, 1970). Later, a group which also included Mechoulam, patented processes for conversion of CBD to either d8THC or d9THC (Webster et al., 2008). In the patented processes, conversion to d8THC was conducted using p-toluenesulfonic acid catalyst with a yield of 81% and high purity, and conversion to d9THC was conducted using boron trifluoride diethyl etherate with a yield of 57% and high purity (Webster et al., 2008).

**FIGURE 5:**
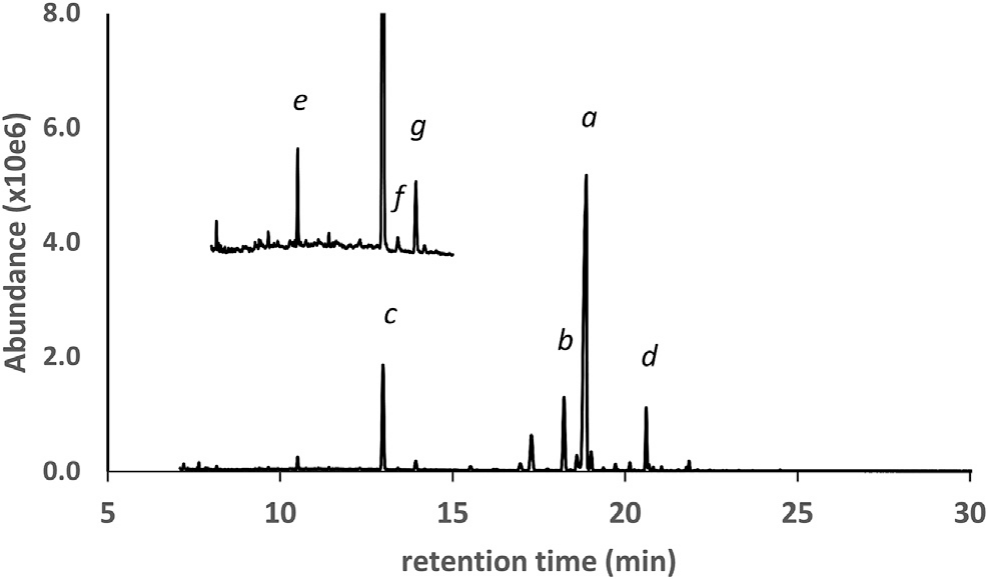
GC-MS chromatogram for a cannabis vaping liquid in which the predominant cannabinoid was d9THC(*a*), and with similar levels of CBD(*c*) and d8THC(*b*). Minor levels of other THC isomers were identified (*not labeled*). CBN(*d*) and the varinols (expanded scale and offset) were also found: CBDV(*e*), d8THCV(*f*), d9THCV(*g*).

**FIGURE 6:**
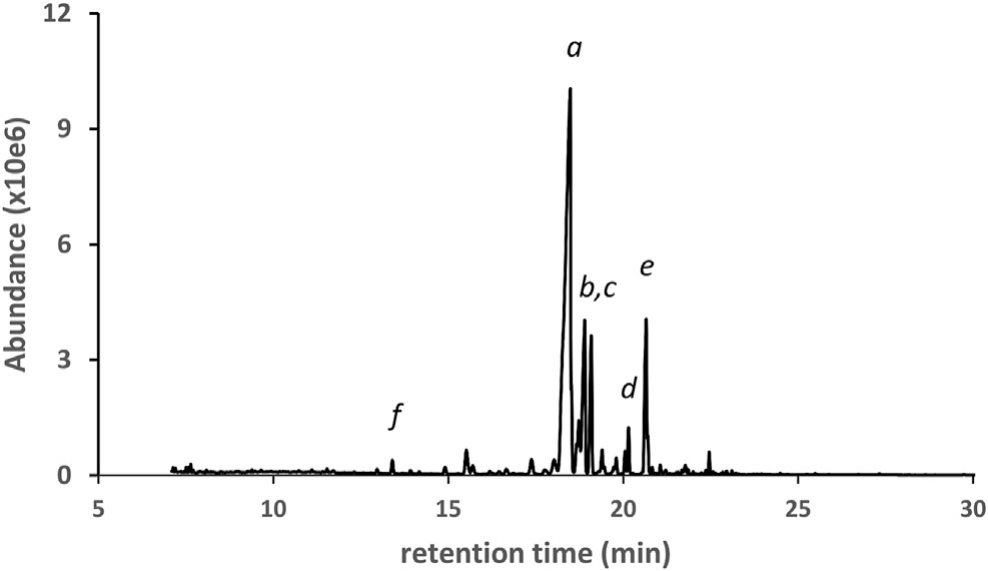
GC-MS chromatogram for a cannabis vaping liquid in which the predominant cannabinoid was d8THC(*a*). d9THC(*b*), d6a,10aTHC(*c*), 6aR,9R-d10THC(*d*), 6aR,9S-d10THC(*not labeled*), and exoTHC(*not labeled*) were also found. Other cannabinoids: CBN(*e*) and d8THCV(*f*).

The next three examples represent vaping liquids in which d9THC, d8THC, and several other THC isomers were found, but with different patterns for the overall profiles of the THC isomers. Figure 5 shows the GC-MS chromatogram for a vaping liquid (VL#6, Table 1) in which the predominant THC isomer was d9THC (peak label *a*) and d8THC (peak label *b*) was second most predominant. The levels of d9THC, d8THC, and CBD (peak label *c*) were determined (HPLC-DAD, %w/w) as 49, 5.4, and 5.2% respectively. Minor levels of three other THC isomers (not labeled in figure) were also identified as follows: d6a,10aTHC, 6aR,9R-d10THC, and 6aR,9S-d10THC. Low levels of the varinols CBDV, d8THCV, and d9THCV (expanded scale and offset, peak labels *e*, *f*, and *g*, respectively), were also found. Figure 6 shows the GC-MS chromatogram for a vaping liquid (VL#8, Table 1) in which the predominant cannabinoid was d8THC (peak label *a*), and substantial levels of both d9THC (peak label *b*) and d6a, 10aTHC (peak label *c*) were also observed. The levels of d8THC and d9THC were determined (HPLC-DAD, %w/w) as 51 and 9.8% respectively. Minor levels of other THC isomers were identified as follows: 6aR,9R-d10THC (peak label *d*), 6aR,9S-d10THC (not labeled in figure), and exoTHC (not labeled in figure).

**FIGURE 7:**
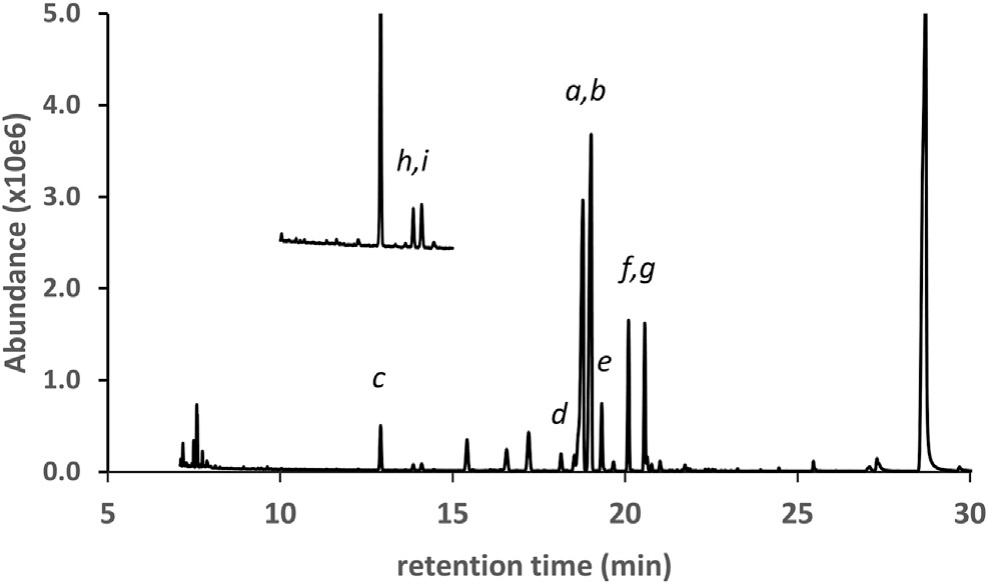
GC-MS chromatogram for a cannabis vaping liquid in which the predominant THC isomers were d9THC(*a*) and d6a,10aTHC(*b*). Additional THC isomers were identified as follows: d8THC(*d*), 6aR,9S-d10THC(*e*), and 6aR,9R-d10THC(*f*). Other cannabinoids include CBD(*c*), CBN(*g*), and the varinols (expanded scale, offset): d9THCV(*h*) and d6a,10aTHCV(*i*).

**FIGURE 8:**
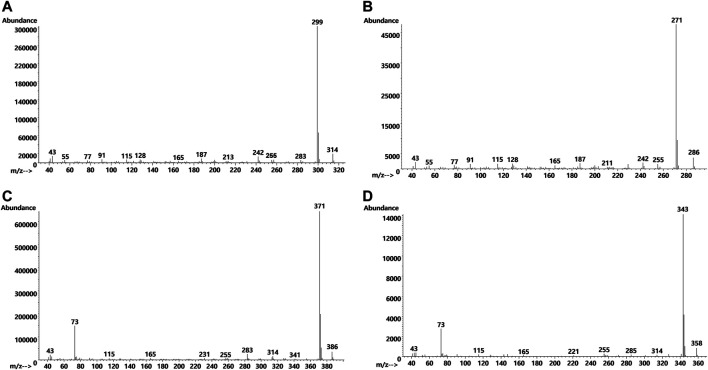
Mass spectra for d6a, 10aTHC parent compound **(A)** and its monoTMS derivative **(C)**, and the analogous spectra for the d6a, 10aTHCV parent compound **(B)** and its monoTMS derivative **(D)**. The d6a, 10aTHC spectra were obtained from a standard, and the d6a, 10aTHCV spectra were obtained from vaping liquids (VL#12–14) in which d6a, 10aTHC was a predominant cannabinoid.

Figure 7 shows the GC-MS chromatogram for a vaping liquid (VL#12, Table 1) in which the predominant THC isomers were d9THC (peak label *a*) and d6a, 10aTHC (peak label *b*). A low level of d8THC (peak label *d*), but marked levels of both 6aR,9S-d10THC (peak label *e*) and 6aR,9R-d10THC (peak label *f*), were also found. The level of d9THC was determined as 11% w/w, but the level of d8THC could not be determined due to a coeluting interferent. Interestingly, both varinols d9THCV and d6a, 10aTHCV were detected (expanded scale and offset, peak labels *h* and *i*, respectively). Parallel conversion of d9THCV to d6a, 10aTHCV would be expected to occur in whatever process caused the conversion of d9THC to d6a,10aTHC. No standard of d6a, 10aTHCV was available for comparison. The identification of d6a, 10aTHCV was based on comparison of its mass spectra with the standard mass spectra for d6a, 10aTHC (Figure 8, comparison of both parent compounds and monoTMS derivatives). The mass spectra for d6a, 10aTHC are unique among all of the THC isomers with fewer high mass ions and overall much less fragmentation both for the parent compound (unit mass 314) and monoTMS derivative (unit mass 386, see Figures 8A,C). The corresponding spectra for d6a, 10aTHCV show the analogous patterns, both for the parent compound (unit mass 286) and monoTMS derivative (unit mass 358, see Figures 8B,D). The elution of d6a, 10aTHCV just after d9THCV also mirrored the elution order for d6a, 10aTHC and d9THC (Figure 7).

The vaping liquids shown in Figures 3-7 represent several different patterns of THC isomer/cannabinoid compositions we encountered. These patterns are evident in Table 1, in which the peak area percentages (PAPs) of selected cannabinoids from the GC-MS chromatograms are summarized. The PAPs represent a good estimate of the relative amounts of the cannabinoids in a given vaping liquid. The selected cannabinoids include all the THC isomers, CBD, and CBN. Both CBD and CBN were included in the peak area percentage (PAP) calculations because they represent possible precursor or degradation products for the THC isomers. CBD amounts may also represent the degree of THC enrichment in the source materials. PAPs for a total of 14 vaping liquids (“VL#”) are listed in the table. Olivetol PAPs are also listed in the table (see last column); however, the olivetol peak areas were not included in the listed PAP calculations for the THC isomers, CBD, and CBN.

VL#1 and VL#2 are vaping liquids in which the only THC isomer detected was d9THC, and d9THC is also the predominant cannabinoid (PAPs near 90%). VL#3 - 5 are vaping liquids in which both d9THC and d8THC are the two predominant THC isomers, with the sum of the PAPs for d9THC and d8THC in the range 80—86%. Although the relative amounts of d9THC and d8THC vary in this group of vaping liquids, they show the same pattern with respect to the other THC isomers. Minor amounts (up to 1.0% PAP) of exoTHC were found. No d6a,10aTHC, nor either of the d10THC stereoisomers, were detected in these vaping liquids. The THC isomer profiles for VL#3 – 5 are similar to the isomer profiles disclosed in patented processes (Grondin and Ward., 2021a and 2021b) for the conversion of either CBD or d9THCA to mixtures of d9THC and d8THC. These processes use heat, and a concentrated or dilute hydrochloric acid catalyst, to effect the cannabinoid conversions on various source materials, which may include isolates, distillates, concentrates, plant extracts, or synthetic sources. The finished products show widely varying ratios of d9THC and d8THC, with minor amounts of exoTHC (Grondin and Ward, 2021b). One further note is that VL#3 - 5 all show the same trend of elevated olivetol levels, with olivetol PAPs (Table 1, third last column) substantially above those for all the other groupings of vaping liquids.

VL#6 and VL#7 are vaping liquids in which d9THC is the predominant cannabinoid (d9THC PAP 66%), with d8THC as the next most prominent (PAPs 11—16%). In these vaping liquids, minor amounts (up to 7% PAP) of the d6a, 10aTHC isomer, and both d10THC stereoisomers, were found. ExoTHC was not detected. VL#8–11 are vaping liquids in which d8THC is the predominant cannabinoid (d8THC PAPs in range 64–79%). In this group of vaping liquids, the next most prominent THC isomers are d9THC and d6a, 10aTHC (combined PAPs in range 9—21%). Minor amounts of both d10THC stereoisomers, and exoTHC were also found. Although the exoTHC isomer was present in minor amounts, this grouping of vaping liquids showed the highest levels of exoTHC. ExoTHC is known to be an impurity associated with dronabinol, a synthetic d9THC (United States Pharmacopoeia, 2008), but is also regarded as an impurity that may be found in Cannabis plant THC isolates (Cid and Van Houten, 2015). This is also the only grouping in which all six of the THC isomers listed in Table 1 were found.

VL#12–14 are vaping liquids in which d9THC and the d6a, 10aTHC are the two predominant THC isomers (combined PAPs all above 70%), with varying ratios of d9THC to d6a,10aTHC. These vaping liquids have the highest amounts of the two d10THC stereoisomers (sum of PAPs in range 10—16%) compared to the other vaping liquid groupings, and also have the lowest amount of d8THC (all PAPs less than 3%) relative to VL#3—11. ExoTHC was not detected. The cannabinoids profiles for VL#12—14 are similar to the profiles disclosed in a US patent (Siegel et al., 2021) for the conversion of d9THC to d10THC, d6a,10aTHC, and CBN. The conversion processes use intact plant materials, THC-sparse oils, or THC-rich oils as starting materials. The reaction conditions include heat and a Lewis acid catalyst such as elemental sulfur. The inventors provide a series of examples in which the finished reaction mixtures contain varying amounts of d9THC, d10THC, d6a,10aTHC, and CBN (Siegel et al., 2021). They further claim to be able to tailor the conditions to maximize or minimize residual d9THC content, and to affect the finished yield ratios of d10THC, d6a,10aTHC, and CBN. As for VL#12—14, the cited examples in the patent contain d9THC, d10THC, and d6a, 10aTHC as the three most predominant THC isomers. Interestingly, d8THC is not reported as present in the finished reaction mixtures, except for one example in which the relative level of d8THC was reported as 0.7% (Siegel et al., 2021). This is also similar to the relative levels of d8THC in VL#12—14, which ranged from 0.4—3.0% (Table 1, PAP results).

The second to last column in Table 1 shows the amount of vaping liquid recovered from each of the 14 vaping devices. Inspection of these results shows that the presence of the THC isomers, and the trends in THC isomer profiles, were not obviously correlated with the extent of vaping liquid consumed, and hence the thermal exposure of the liquid during the vaping process. For example, VL#2 represents a vaping liquid in which over 95% of the cartridge contents were consumed, yet no unnatural THC isomers were detected. The trends for both the VL#8—11, and VL#12–14 groupings, each include vaping liquids which came from unused or minimally used cartridges, as well as almost empty cartridges. This is not to conclude that the vaping process and exposure of the vaping liquid to the vaporizer heating elements cannot alter the vaping liquid composition. It is important to note that the vaping products in this work represent several different types of vaping devices. Different vaping devices may alter vaping liquid compositions to varying degrees, and this aspect is not studied here. The last column in Table 1 shows the additives identified in the vaping liquids according to the protocol described in Part 2^1^. Again, there is no obvious correlation between the THC isomer profiles and the additive types.

As discussed above, the THC isomer profiles for the VL#3 – 5 grouping show similarities to the reported isomer profiles for THC source materials produced by one particular commercial process (Grondin and Ward, 2021a and 2021b). In addition, the THC isomer profiles for the VL#12—14 grouping are similar to the reported isomer profiles of THC source materials produced by yet another distinct commercial process (Siegel et al., 2021). The trends in THC isomer profiles suggest that the vaping liquids were likely formulated with THC source materials which already contained unnatural THC isomers. In the course of our work with the vaping liquids, we received two different bulk THC distillates (“DST#1” and “DST#2”). The THC distillates represent examples of high THC source materials prior to incorporation into a vaping liquid composition, and thus provide data on THC isomer profiles associated with the manufacturing process. The specifics of the processes used to manufacture these distillates are not known.

**FIGURE 9:**
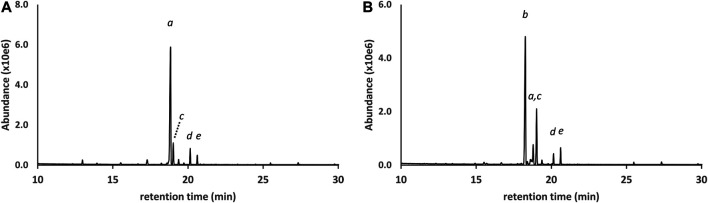
GC-MS chromatograms for two different bulk THC distillates in which the primary cannabinoid was either d9THC **(A)** or d8THC **(B)**. Peak labels d9THC(*a*); d8THC(*b*); d6a,10aTHC(c); 6aR,9R-d10THC(*d*); CBN (*e*). Lower levels of additional THC isomers (*not labeled*) were also identified in both distillates. See text for discussion.

GC-MS analysis for DST#1 (Figure 9A and Table 1) showed that the primary cannabinoid was d9THC, with all of the other THC isomers present except for exoTHC. The d9THC level in DST#1 was 60% w/w (HPLC-DAD), but the level of d8THC could not be determined due to a coeluting interferent. The THC isomer profile for DST#1 resembles the profiles for the grouping of vaping liquids VL#12—14, and the Siegel process (Siegel et al., 2021). GC-MS analysis for DST#2 (Figure 9B and Table 1) showed that the primary cannabinoid was d8THC, with all of the other THC isomers present. The d8THC and d9THC levels in DST#2 were 53 and 6.2% w/w, respectively (HPLC-DAD). The THC isomer profile for DST#2 resembles the profiles for the grouping of vaping liquids VL#8–11. The results for these THC distillates confirm that some manufacturing processes produce unnatural THC isomers at significant levels. The similarities between the THC distillates and the vaping liquids provides further evidence that the vaping liquids were likely formulated with Cannabis source materials which already contained unnatural THC isomers, whether or not further changes in composition occurred during the vaping process.

Additional distinct commercial scale processes have been reported for the production of THC source materials which contain unnatural THC isomers. Tegen and Cho have reported the use of p-toluenesulfonic acid catalyst to convert CBD to mixtures of d8THC and d9THC, or convert CBD to mainly d8THC or mainly d9THC (Tegen and Cho., 2019a; 2019b). The conversion processes may be conducted starting with pure CBD or hemp extracts. The inventors provided examples with crude product relative amounts of 1.1% CBD, 18.4% d8THC, 80.0% d9THC, or 96.5% d8THC, 2.5% d9THC, from pure CBD starting materials. A third example had crude product relative amounts of 2% d8THC and 98% d9THC from hemp extract starting materials.

A recent world patent application (Hartman, 2020) reports on commercial processes for the conversion of either CBD or d9THC to various combinations of d8THC, d10THC, and cannabinol. Cited starting materials include hemp isolates, marijuana isolates, CBD isolates, and hexane or butane extracts of hemp or marijuana. The isolates are obtained via CO_2_ supercritical fluid extraction. Conversions are effected using heat, and may include halogen or free radical generator catalysts such as iodine. Specified reaction temperatures can range from 55—800°C. The disclosed reaction scheme shows conversion of CBD to d9THC, followed by conversion of d9THC to d8THC, d10THC, and cannabinol. Relative conversion yields of CBD to d8THC in the range 50–99% are claimed, but no conversion yields for d10THC were cited. The parallel conversions of some varinol cannabinoids such as conversion of CBDV to d9THCV, with subsequent conversion of d9THCV to d8THCV, are also disclosed.

For all of the vaping liquids and THC distillates, a similar pattern with respect to the relative isomer amounts of d6a,10aTHC, 6aR,9R-d10THC, and 6aR,9S-d10THC was observed. The amount of d6a, 10aTHC was always higher than either of the two d10THC isomers, and the amount of the 6aR,9R-d10THC isomer was always higher than the amount of the 6aR,9S-d10THC isomer. These results suggest preferential formation within this set of isomers. The formation of 6aR,9R-d10THC and 6aR,9S-d10THC from d9THC and/or d8THC under base-catalyzed conditions was reported by Srebnik et al. (1984), with the 6aR,9R-d10THC isomer being thermodynamically favored. Further isomerization of the d10THC isomers to the d6a, 10aTHC isomers was carried out under acid-catalyzed conditions (Srebnik et al., 1984), with 6aR,9R-d10THC forming the 9R-d6a, 10aTHC enantiomer and 6aR,9S-d10THC forming the 9S-d6a, 10aTHC enantiomer.

It is also of note that olivetol, which represents a structural piece (see Figure 1, lowest right structure) of the THC molecule, was only detected in vaping liquids and THC distillates in which unnatural THC isomers were also present. Olivetolic acid (the carboxylated form of olivetol), is known to be a key compound in the plant biosynthesis of the cannabinoids (Hanuš et al., 2016), and olivetol is used as a starting material in the classical synthesis of d9THC (Bloemendal et al., 2020). The significance of the olivetol finding in the vaping liquids is not clear. However, the olivetol peak area percentages appear to vary among the different vaping liquid groupings, but are rather consistent within each vaping liquid grouping (Table 1). This suggests some relation to the THC source materials, such as varying degrees of concentration of the plant constituents during purification steps, or even varying degrees of degradation of the cannabinoids among distinct chemical and/or thermal treatments. However, we could find no relevant literature to address this question.

It is not our intention to present the GC-MS peak area percentage (PAP) results for the THC isomers in the vaping liquids and distillates (Table 1) as strict quantitative values. Rather, the GC-MS PAP results are considered reasonable estimates of the relative amounts of the THC isomers, and are supported by the HPLC-DAD analysis. For reasons stated earlier, HPLC-DAD quantitative results were only obtained for the d9THC and d8THC isomers. Table 2 shows a comparison of GC-MS PAPs and HPLC-DAD quantitative analysis (%w/w) for d9THC and d8THC in the vaping liquids and distillates from Table 1. To allow for a more direct comparison of the GC-MS and HPLC-DAD results, the ratios of d9THC:d8THC were calculated for both the GC-MS PAP data, and the HPLC-DAD % w/w data (Table 2, columns 4 and 7, respectively). Inspection of the d9THC:d8THC ratio data columns shows a strong correlation between the GC-MS and HPLC-DAD results. The d9THC:d8THC PAP ratios agree with the d9THC:d8THC %w/w ratios by factors which range from 0.7—1.4 (factors calculated as the ratio of the PAP and %w/w ratios). Although quantitation was not obtained for the other THC isomers identified in the GC-MS analysis, their presence was confirmed in the HPLC-DAD analysis of the vaping liquids and distillates by retention time and UV spectral matches with the qualitative reference standards; this applies to the exoTHC, d10THC, and d6a, 10aTHC isomers.

**TABLE 2:** Comparison of GC-MS peak area percentages (PAP) and HPLC-DAD quantitative analysis (%w/w) for d9THC and d8THC in vaping liquids and bulk THC distillates.

	GC-MS analysis			HPLC-DAD analysis		
Vaping			PAP ratio			%w/w ratio
Liquids	d9THC PAP	d8THC PAP	d9THC:d8THC	d9THC %w/w	d8THC %w/w	d9THC:d8THC
VL#1	89.2	ND	NA	78.2	ND	NA
VL#2	93.7	ND	NA	77.1	ND	NA
VL#3	44.2	35.5	1.2	17.4	10.5	1.6
VL#4	16.9	69.2	0.24	5.40	16.9	0.32
VL#5	16.2	66.3	0.24	7.41	21.1	0.35
VL#6	65.8	10.4	6.3	48.8	5.36	9.1
VL#7	65.5	15.6	4.2	44.7	7.78	5.7
VL#8	11.8	63.8	0.18	9.80	51.2	0.19
VL#9	9.49	68.8	0.14	5.35	52.4	0.10
VL#10	6.06	72.0	0.084	3.77	35.2	0.11
VL#11	1.41	79.0	0.018	0.993	58.6	0.017
VL#12	33.0	1.42	23	10.9	^a^	^a^
VL#13	51.1	2.96	17	19.9	0.88	23
VL#14	43.1	0.439	98	11.5	^a^	^a^
DISTILLATES						
DST#1	76.0	0.683	111	60.4	^a^	^a^
DST#2	7.75	60.4	0.13	6.19	53.1	0.12

a The d8THC levels in these items was observed to be low in the HPLC-DAD analysis, but accurate quantitation.

was not possible due to a coeluting interferent.

ND-not detected; NA-not applicable.

## Concluding Remarks

While the grouping of vaping liquids presented in this work is somewhat subjective, this work provides strong evidence that different THC isomer patterns observed in the products represent different processes to produce high THC source materials. The vaping liquids reported in this work were obtained from multiple locations throughout the US, showing that the occurrence of unnatural THC isomers in these products was widespread. Given that these isomers are likely already present in high THC source materials, it is to be expected that unnatural THC isomers will be encountered in other THC containing products. We recently encountered some THC candies in which both d9THC and d6a, 10aTHC were predominant cannabinoids, and significant amounts of d8THC and both d10THC stereoisomers were found (unpublished data). The presence of unnatural THC isomers in Cannabis products raises questions with respect to both their legality and potential safety concerns. All of the THC positional isomers and their stereochemical variants are listed as Schedule 1 in the 1971 Convention on Psychotropic Sustances (WHO Expert Committee on Drug Dependence, 2018b), and by the US DEA (US Drug Enforcement Administration (DEA) Department of Justice, 2020c). Many of the state laws in the US only address the d9THC isomer, leaving much ambiguity. With regard to the pharmacological effects and safety of the other THC isomers, only the d8THC isomer has been studied to some extent (Hollister and Gillepsie 1973; Thomas et al., 1990; Punyamurthula et al., 2017; Thapa et al., 2018), and we are not aware of any published reports for the other THC isomers.

## Data Availability

The datasets presented in this article are not readily available because data may be considered sensitive or require a special request for public disclosure. Requests to access the datasets should be directed to laura.ciolino@fda.hhs.gov.

## Footnotes

1 Ciolino, L. A., Falconer, T. M., Ranieri, T. L., Brueggemeyer, J. L., Taylor, A. M., and Mohrhaus, A. S. (2021). EVALI Vaping Liquids Part 2: Mass Spectrometric Identification of Diluents and Additives. Front. Chem. (Under review)
